# Real-Time Φ-OTDR Vibration Event Recognition Based on Image Target Detection

**DOI:** 10.3390/s22031127

**Published:** 2022-02-02

**Authors:** Nachuan Yang, Yongjun Zhao, Jinyang Chen

**Affiliations:** 1 Data and Target Engineering Institute, PLA Strategic Support Force Information Engineering University, Zhengzhou 450001, China; zhaoyongjuntg@126.com (Y.Z.); jinyang870210@163.com (J.C.); 2 Research Institute for National Defense Engineering of Academy of Military Science PLA, Luoyang 471023, China

**Keywords:** phase-sensitive optical time-domain reflectometer, computer vision, target detection, real-time processing

## Abstract

Accurate and fast identification of vibration signals detected based on the phase-sensitive optical time-domain reflectometer (Φ-OTDR) is crucial in reducing the false-alarm rate of the long-distance distributed vibration warning system. This study proposes a computer vision-based Φ-OTDR multi-vibration events detection method in real-time, which can effectively detect perimeter intrusion events and reduce personnel patrol costs. Pulse accumulation, pulse cancellers, median filter, and pseudo-color processing are employed for vibration signal feature enhancement to generate vibration spatio-temporal images and form a customized dataset. This dataset is used to train and evaluate an improved YOLO-A30 based on the YOLO target detection meta-architecture to improve system performance. Experiments show that using this method to process 8069 vibration data images generated from 5 abnormal vibration activities for two types of fiber optic laying scenarios, buried underground or hung on razor barbed wire at the perimeter of high-speed rail, the system mAP@.5 is 99.5%, 555 frames per second (FPS), and can detect a theoretical maximum distance of 135.1 km per second. It can quickly and effectively identify abnormal vibration activities, reduce the false-alarm rate of the system for long-distance multi-vibration along high-speed rail lines, and significantly reduce the computational cost while maintaining accuracy.

## 1. Introduction

Phase-sensitive optical time-domain reflectometer (Φ-OTDR) is a simple and effective method to measure single-mode fiber vibration [1,2], which has the advantages of distributed detection capability up to 100 km at a single station [3], as well as high resolution, anti-electromagnetic interference, corrosion resistance, low energy loss, flame and explosion resistance [4], and can be combined with Raman pumping amplification to achieve long-range distributed abnormal vibration location inspection [5], which makes Φ-OTDR extensively employed in perimeter security and oil pipeline monitoring.

However, the long-range detection and high sensitivity of Φ-OTDR will inevitably result in high nuisance-alarm rates (NARs). In the case of vibration detection along the fence and the ground near the fence of the high-speed railway (HSR) at this study, noise mainly comes from the frequency drift and linewidth range of the optical devices, thermal noise, and scattering noise of electronic devices. The interference signals are caused by the airflow in the detection environment. Based on the hardware realization of long-distance vibration detection, the subsequent signal processing and vibration classification problems become the essential factors for Φ-OTDR to be able to identify abnormal vibration signals precisely, and the precision of the identification significantly affects the eventual false alarm probability of the system. For the nonstationary vibration signals detected by Φ-OTDR, wavelet noise reduction can be used for long-range slow time-domain perturbation signal purification to achieve vibration localization [5]. Using wavelet packet decomposition to extract the features of each energy layer of vibration signal, combined with an artificial neural network classifier, high-precision recognition for vibration signals can be achieved [6]. Short-time Fourier transform is also a typical feature extractor, and combined with a Gaussian mixture model or convolutional neural network can complete distinct intrusion activity detection and classification [7,8].

The above identification methods extract the slow time-domain signal features of a vibration location and then distinguish various vibration activities by a well-designed classifier. Nevertheless, since the fiber strain and refractive index near the vibration activity will vary, it is complicated to determine the vibration source location in practical engineering applications. The deviation of vibration source location selection will also decrease the recognition rate. In contrast, morphological feature extraction based on spatio-temporal domain images can improve the recognition rate by adding contextual features [9]. When the morphologic features of time-space domain signals are extracted and obtained in the experiment, the relevance vector machine (RVM) can be used to achieve a relatively short recognition time of below 1 s for vibration behavior [10]. In addition, convolutional neural networks (CNNs) can effectively classify a single vibration activity image without expert knowledge [11,12].

For 100-km level detection, however, various vibration activities may occur simultaneously in a single spatio-temporal domain image, and the real-time capability of the system cannot be guaranteed on account of the apparent increment in total recognition time. This problem necessitates the detection of multiple vibrational activity classes in a single vibration alarm event spatio-temporal image by the target detection algorithm of computer vision. Convolutional neural network-based target detectors are distinguished into two categories: region-based CNNs (R-CNNs) [13] and their variants [14,15] belong to two-stage target detectors, which utilize the region-based proposal sliding window method to monitor targets in images; in contrast to two-stage detectors, single-step detectors, such as You Only Look Once (YOLO) detectors [16] and their variants [17,18,19], employ down-sampling components and concatenation components or a path aggregation network to accomplish feature extraction of the original image in different dimensions, directly mapping from feature maps in different sizes to target probabilities, bounding box locations, and target classification probabilities, which can increase the number of detected frames per second with comparable performance [19]. YOLO detectors are the State-Of-The-Art (SOTA) object detection algorithms in deep learning [20,21], and are real-time and efficient in practical applications for portable embedded devices and have significant advantages in terms of accuracy and processing speed [22]. While the feature extraction network in the existing YOLO detection framework is designed for publicly available target detection datasets [23,24], the corresponding feature extraction network needs to be improved for a customized dataset.

Considering the Φ-OTDR high-speed railway perimeter security detection environment, this paper aims to complete monitoring of multiple vibration images in finite time and complete multiple vibration target identification and localization in single image detection. High-speed rail perimeter security is mainly threatened by climbing or jumping over the fence. Early warning threats include people walking on the ground near the fence, manual excavation, construction machinery, and interference, primarily from the shaking of optic fiber caused by airflow due to natural wind or high-speed rail. Single-mode optical fiber is laid on the razor barbed wire on the fence of the high-speed railway station to detect the vibration signals of people climbing or jumping over the fence and the shaking caused by airflow. About 1 km long single-mode optical fiber is buried at 100 m from the initial fiber position and 20 cm underground to detect the early warning activities of people walking, manual excavation, and mechanical construction close to the fence. The raw signals acquired by Φ-OTDR are purified by signal pre-processing and image pre-processing to form the ultimate dataset. An improved YOLO model is trained and evaluated to accurately and quickly recognize abnormal vibration events. The contributions of this study are: (1) The effect of parameter adjustment in the signal pre-processing module on inspection is illustrated from the perspective of statistical theory; (2) An improved YOLO model for high-speed railway perimeter abnormal vibration detection is proposed to achieve single detection of multiple vibration targets; (3) The effect of different feature extraction networks on the detection performance of the improved YOLO model is tested and discussed; (4) The detection performance of the improved YOLO model with other State-of-The-Art (SOTA) models has been compared and summarized.

This paper is organized as follows: Section 2 describes the entire system structure and analyzes the statistical characteristics of the raw signal acquired by Φ-OTDR. Section 3 presents the principle of demodulating vibration signals from raw signals and proposes signal pre-processing methods to improve the signal-to-noise ratio (SNR) and detection probability, thereby illustrating the signal pre-processing procedure. Sequentially, it introduces the way to acquire vibration data and generate the spatio-temporal images to be identified. Section 4 discusses the experimental dataset generation method and the components of the multi-target detection system, the effects of different feature extraction network components on the detection performance of the data images, the basis for model improvement, and the evaluation metrics. Section 5 focuses on the experimental procedure, examines and compares the impact of different feature extraction networks on recognition, and compares the performance of the improved YOLO model with other SOTA models. The last section concludes the paper.

## 2. Baseline System

### 2.1. System Overview

For the high-speed railway perimeter detection environment with many noise and interference signals, to quickly and accurately identify the vibration source class and thus reduce the false-alarm rate, it is necessary first to design the signal pre-processing algorithm and set the corresponding signal acquisition parameters based on the optical pulse detection principle. Up to this point, the system has removed most of the noise to improve the SNR of the Φ-OTDR detection signal and effectively detect vibration activities. In practice, airflow can generate interference vibration signals, so such interference signals need to be effectively identified and excluded from the necessary alarm to improve the reliability of the overall system alarm. In this regard, it is essential to generate images from the pre-processed data matrix, enhance the vibration target features, and finally use an improved image target detection algorithm to identify all classes of vibration activities accurately.

**Figure 1:**
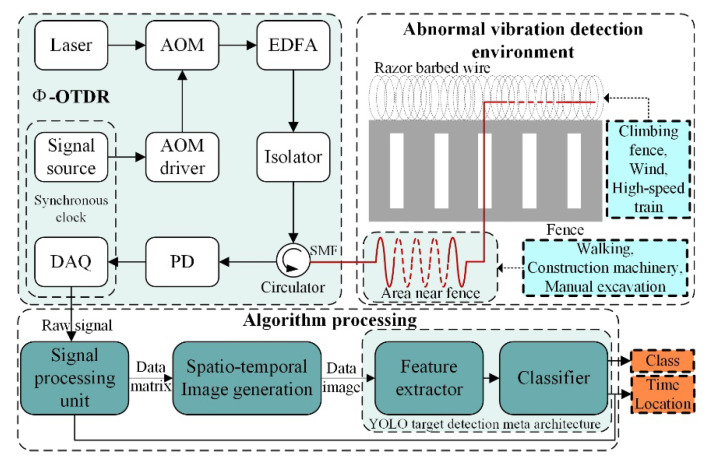
Overall system operation structure.

As shown in Figure 1, the real-time Φ-OTDR vibration events detection system contains three components: (1) Abnormal vibration detection environment, which is the environment for abnormal vibration detection at the perimeter of the high-speed railway. The primary monitoring events are the five kinds of vibration signals mentioned above. The single-mode optical fiber is laid in the detection environment by burying underground or hanging on the razor barbed wire to realize the distributed real-time detection of abnormal vibration. (2) In the Φ-OTDR system, light probe pulses generated by a coherent laser source with narrow linewidth are injected into the single-mode fiber through an optical circulator. Due to the elastic scattering effect, the backscattered light interferes with each scattering center in the fiber to generate Rayleigh backscattered light. The backscattered light is converted into a digital signal by the data acquisition (DAQ) card through the photodetector (PD). Then, the digital signal is transmitted to the algorithm processing module. (3) The algorithm processing module functions as follows:

1. The original digital signal is processed by the signal processing unit to accomplish pulse accumulation, pulse cancellers, maximum value processing to generate the primary data matrix, and then determine the location of the vibration signal appearing on the detection fiber and the corresponding time according to the statistical detection principle;
2. On this basis, the data matrix is converted into grayscale images and then processed by median filtering and pseudo-color to generate the final spatio-temporal image of vibration data to be detected;
3. The images are detected by the improved YOLO target detector, which first completes the multi-scale feature conversion by the feature extraction network, then uses the classifier to achieve the detection of multiple vibration targets in a single image, and eventually improves the detection accuracy and reduces the false-alarm rate of the system.

In this application, the raw signal acquired by Φ-OTDR is processed by the signal processing unit and spatio-temporal image generation module to generate a customized dataset. The dataset is subsequently divided into the training set, validation set, and test set. The training and validation sets are employed to train and modify the improved YOLO target detection model. The test set is applied to test and evaluate the model performance, then select the optimal model.

On this foundation, the spatio-temporal image, which is generated from real-time vibration data, is transmitted to the improved optimal model to obtain the classes of multiple vibration activities and their locations in the image, thereby reducing the false-alarm rate and ensuring the real-time effectiveness of the distributed fiber optic perimeter security system.

### 2.2. Φ-OTDR Raw Signal Statistical Characteristics

**Figure 2:**
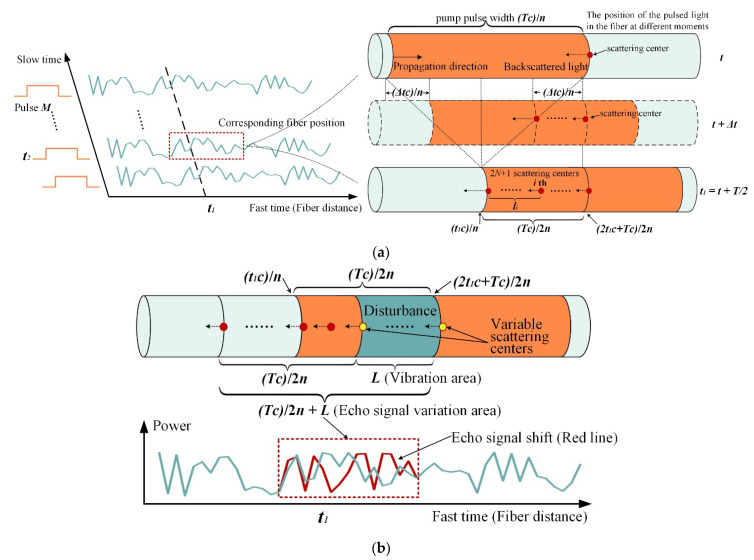
Φ-OTDR backscattering principle: (**a**) Superposition principle of backscattered light from all scattering centers within a half-light pulse width; (**b**) The effect of vibration on the echo signal, where the variable scattering center is marked in yellow.

The signal processing unit utilizes the statistical hypothesis detection principle to obtain the location and corresponding time of all vibration events on the detection fiber, and uses signal processing methods to improve the signal-to-noise ratio to enhance the accuracy and precision of subsequent pattern recognition. Φ-OTDR backscattered light is generated by the backscattering interference of coherent pulsed light from 2N +1, elastic scattering centers in the region covered by half of the optical pulse width; refer to Figure 2a.

Define the attenuation coefficient of the fiber as α, after Acousto-optic Modulator (AOM) modulation and Erbium Doped Fiber Amplifier (EDFA) amplification, a sequence of coherent optical pulses of periodic width $\left(Tc\right)/n$ is injected into a single-mode fiber with group refractive index n, the optical field is $A_{0}exp(j\left(2\pi Ft_{1}+\theta \left(t_{2}\right)\right))$, $t_{2}$ records the slow-time domain pulse emission time, and pulse repetition interval (PRI) indicates the difference in emission time of neighbor pulses. The specific backscattering process can be analyzed from the change in position of the light pulse on the fiber at different moments in a single detection.

Considering $t_{2}$ as the starting time, the backscattered light from the light pulse at the moment $t_{1}$ in the fast-time domain is converted into an electrical signal by a PD, and then quantized and saved by a DAQ. The integrated reflection coefficient σ at position $\left(t_{1}c\right)/n$ is derived from the combined effect of the 2N +1 scattering centers in range $l\left(t_{1}\right)\in \left[\left(t_{1}c\right)/n,\left(2t_{1}c+Tc\right)/2n\right]$. Specifically, at the moment t, the central position of the light pulse propagates to $\left(t_{1}c\right)/n$, and the backscattered light from the scattering center at $\left(2t_{1}c+Tc\right)/2n$ begins to scatter in the opposite direction of the light pulse propagation. t+Δt moment, the above backscattered light propagates to $\left(2t_{1}c+Tc-2\Delta tc\right)/2n$, during which the backscattered light is the superposition of a series of small Rayleigh scattering centers within Δtc/n. When the pulsed light propagates half the light pulse width, i.e., the tail end of the pulsed light propagates to $\left(t_{1}c\right)/n$, the backscattered light at this point is superposed by the backscattered light from 2N +1 scattering centers at different spatial positions within half the light pulse width $l\left(t_{1}\right)$, and there is no further superposition. In the range of $l\left(t_{1}\right)$, the reflectivity corresponding to the ith scattering center is $\sigma_{i}$, the distance to the $\left(t_{1}c\right)/n$ position is $l_{i}$, the echo phase is $exp(j\left(2\pi F\left(t_{1}-2nl_{i}/c\right)+\theta \left(t_{2}\right)\right))$, and $\theta \left(t_{2}\right)$ indicates the initial phase of the pulsed light. The final superposed echo signal $\overset{⎯}{\zeta }\left(t_{1},t_{2}\right)$ at the $\left(t_{1}c\right)/n$ position can be expressed as:

(1) $$\begin{array}{ll} \overset{⎯}{\zeta }\left(t_{1},t_{2}\right) & =A_{0}\sum_{i=-N}^{N}\sqrt{\sigma_{i}}exp\left(-\alpha l_{i}\left(t_{1}\right)\right)exp\left(j\left(2\pi F\left(t_{1}-2nl_{i}\left(t_{1}\right)/c\right)+\theta \left(t_{2}\right)\right)\right)\\ & =A_{0}exp(-\alpha \bar{l\left(t_{1}\right)})exp(j\left(2\pi Ft_{1}+\theta \left(t_{2}\right)\right))\sum_{i=-N}^{N}\sqrt{\sigma_{i}}exp(-j4\pi nl_{i}/\lambda ) \end{array}$$

where λ represents light wavelength. Equation (1) reveals that the relative positions $l_{i}$ of all scattering centers in the half-wavelength region are interrelated with the echo signal $\overset{⎯}{\zeta }\left(t_{1},t_{2}\right)$. In the presence of vibrations in the area, the vibration-induced fiber strain leads to changes in $l_{i}$ and refractive index n at different positions, which in turn cause shifts in the backscattered light phase and eventually change $\overset{⎯}{\zeta }\left(t_{1},t_{2}\right)$. As demonstrated in Figure 2b, both the variation of the relative position of the scattering center within the half-light pulse width $l\left(t_{1}\right)$ and the change of the refractive index n lead to the shift of the echo signal at $\left(t_{1}c\right)/n$. Furthermore, when the scattering centers within the vibration area L are disturbed, the vibration interferes with the backscattered light phase, leading to a shift in the echo signal within the range $\left(Tc\right)/2n+L$. The location of the vibration occurrence is judged by detecting the area where the echo signal shift is located.

For analysis of the echo signal, define $\xi =\left|exp(j\left(2\pi Ft_{1}+\theta \left(t_{2}\right)\right))\sum_{i=-N}^{N}\sqrt{\sigma_{i}}exp(-j4\pi nl_{i}/\lambda )\right|=\left|\sqrt{\sigma \left(t_{1},t_{2}\right)}exp(-j\phi \left(t_{1},t_{2}\right))\right|$, $\phi_{i}=4\pi nl_{i}/\lambda$. According to Euler’s formula, the integrated reflection coefficient $\sigma \left(t_{1},t_{2}\right)$ and the integrated phase fluctuation $\phi \left(t_{1},t_{2}\right)$ can be expressed as:

(2) $$\left\{\begin{array}{c} \sigma \left(t_{1},t_{2}\right)={\left(\overset{N}{\underset{i=-N}{\sum }}\sqrt{\sigma_{i}}\;\sin\left(\phi_{i}\right)\right)}^{2}+{\left(\overset{N}{\underset{i=-N}{\sum }}\sqrt{\sigma_{i}}\cos\left(\phi_{i}\right)\right)}^{2}\\ \phi \left(t_{1},t_{2}\right)=\arctan(\overset{N}{\underset{i=-N}{\sum }}\sqrt{\sigma_{i}}\;\sin\left(\phi_{i}\right)/\overset{N}{\underset{i=-N}{\sum }}\sqrt{\sigma_{i}}\cos\left(\phi_{i}\right)) \end{array}\right.$$

According to the central limit theorem, the real and imaginary parts of $\sum_{i=-N}^{N}\sqrt{\sigma_{i}}exp(-j4\pi nl_{i}/\lambda )$ are statistically independent Gaussian random variables, and the echo phase is uniformly distributed, so ξ is Rayleigh distributed, and $\sigma \left(t_{1},t_{2}\right)$ is exponentially distributed [25]. It is observed that the phase jitter $\theta \left(t_{2}\right)$ of the pulsed light at different moments does not affect the final acquisition results, avoiding the effect of phase noise introduced by the laser and the AOM [26].

The photodetector output electrical signal can be expressed as the following Equation:

(3) $$I\left(t_{1},t_{2}\right)=\rho \overset{⎯}{\zeta }{\overset{⎯}{\zeta }}^{*}=\rho A_{0}^{2}e^{-2\alpha \bar{l(t_{1})}}\xi^{2}=\rho A_{0}^{2}e^{-2\alpha \bar{l(t_{1})}}\sigma \left(t_{1},t_{2}\right)$$

where the photoelectric conversion rate ρ, the initial optical power $A_{0}^{2}$ and the light decay rate α can be considered as fixed values, while the variation of the collected vibration data is from the variation of the integrated reflection coefficient $\sigma \left(t_{1},t_{2}\right)$. The electrical signals received by the DAQ in the absence of vibration signals are the coherent noise power at each location along the fiber.

## 3. Raw Signal Pre-Processing and Image Generation

### 3.1. Analysis of Vibration Detection Principles

According to the analysis in Section 2.2, the real and imaginary parts of the optical echo signal z at the position $\left(t_{1}c\right)/n$ of the fiber can be considered as statistically independent zero-mean Gaussian random variables when the fiber is devoid of vibration in the range $l\left(t_{1}\right)$. The backscattered signal z is the sum of the Gaussian white noise sample w and the target component s in the presence of a vibrational signal in this range, where s is a complex constant of unknown amplitude $\sqrt{\sigma_{s}}$ and phase $\phi_{s}$, $s=\sqrt{\sigma_{s}}exp(-j\phi_{s})$, during one sweep. The signal z received by the photodetector in one inspection decision can be expressed as the following two hypotheses:

(4) $$\begin{array}{c} H_{0}:z=w\;\;\;\;w\sim CN\left(0,\delta_{w}^{2}\left(t_{1}\right)/2\right)\\ H_{1}:z=s+w\;\;\;\;\;\;\;\;\;s=\sqrt{\sigma_{s}}e^{-j\phi_{s}} \end{array}$$

where $\delta_{w}^{2}\left(t_{1}\right)$ is related to the coherent noise at $\left(t_{1}c\right)/n$. The null hypothesis $H_{0}$ indicates that there is no vibration event within the corresponding detection location, and the probability density function (PDF) of $y=\left|z\right|$ is Rayleigh distributed. The PDF of y in the alternative hypothesis $H_{1}$ is the Rice distribution, cf. Equation (5), where $I_{0}\left(\cdot \right)$ is the modified Bessel function of the first kind:

(5) $$p_{y}\left(y|\sqrt{\sigma_{s}},H_{1}\right)=\left\{\begin{array}{cc} \frac{2y}{\delta_{w}^{2}}exp\left(-\frac{\left(y^{2}+\sigma_{s}\right)}{\delta_{w}^{2}}\right)I_{0}\left(\frac{2\sqrt{\sigma_{s}}y}{\delta_{w}^{2}}\right) & y\geq 0\\ 0 & y<0 \end{array}\right.$$

For a multi-pulse sampling system with a noncoherent integration of M samples, a modified Bessel function of the first kind approximation leads to the statistic y′, where λ is the detection threshold satisfying a fixed false-alarm rate:

(6) $$y^{\prime }=\overset{M-1}{\underset{m=0}{\sum }}y_{m}^{2}\begin{array}{c} H_{1}\\ ≷\\ H_{0} \end{array}\frac{\delta_{w}^{4}}{\sigma_{s}}\left(\ln(\lambda \right)+\frac{\sigma_{s}}{\delta_{w}^{2}})=\lambda^{\prime }$$

The presence of a vibration activity is detected by determining whether the sum of the squares of the acquired signal amplitudes, $y^{\prime }$, exceeds the threshold $\lambda^{\prime }$. If $H_{0}$ best accounts for the data, and the false alarm probability $P_{\mathrm{FA}}$ is a fixed value, the threshold $\lambda^{\prime }$ is only related to the number of noncoherent integration samples, M, and coherent noise $\delta_{w}^{2}$. If $H_{1}$ best accounts for the data, the integrated reflection coefficient $\sqrt{\sigma_{s}}$, which is independent in all M samples of a single detection decision, shows different distribution characteristics $p_{\sigma }\left(\sigma_{s}\right)$ for different vibration sources [7,27]. The PDFs of the final statistic $y^{\prime }$ corresponding to the different vibration modes can be found using characteristic functions. The detection probability of different vibrations is the integral value of the corresponding PDF within the range exceeding the threshold.

Either improving the system signal-to-noise ratio or increasing the noncoherent integration samples number M can lead to an increase in the detection probability $P_{D}$ of the system for vibration signals. High-frequency noise can be suppressed by pulse cancellers to improve the system detection probability from the perspective of improving the signal-to-noise ratio. In addition, increasing the noncoherent integration samples number M can improve the detection performance with a constant SNR.

### 3.2. Signal Pre-Processing Procedure

In the signal acquisition process, there are $M_{p}$ detection pulses contained within one detection sweep. The backscattered light is converted to an electrical signal I after PD, and the detection data collected by DAQ at the fixed position $\left(t_{1}c\right)/n$ for a single pulse is $x=I\left(t_{1},t_{2}\right)=\rho A_{0}^{2}exp\left(-2\alpha \bar{l\left(t_{1}\right)}\right)\sigma \left(t_{1},t_{2}\right)$. An array of detection data $X=\left[x_{0},\;x_{1},\cdots x_{m},\cdots x_{M_{p}-1}\right]$ can be obtained at the fixed fiber position in one detection sweep. The number of noncoherent integration samples is M, the pulse canceller interval is $M_{g}$, and detection data array is converted into $M_{c}=M_{p}-M-1-M_{g}$ statistics $X^{\prime }=\left[x_{0}^{\prime },\;x_{1}^{\prime },\cdots x_{i}^{\prime },\cdots x_{M_{c}-1}^{\prime }\right]$, where:

(7) $$x_{i}^{\prime }=\sum_{m=i}^{M-1+i}(x_{m}-x_{m+M_{g}}),\;i\in \left[0,M_{c}-1\right]$$

as the final detection value $x_{max}^{\prime }=\max\left(X^{\prime }\right)$ is taken and different threshold values are set at different locations. The vibration signal is considered to exist if $x_{max}^{\prime }$ exceeds the threshold. The above steps are repeated for different detection locations within one sweep, thus the vibration detection is completed from $M_{p}$ pulses to one whole fiber. Moreover, to improve the reliability of the detection decision, the decision rule is defined as a vibration activity being detected at least K times in multiple decisions before it is finally determined as a valid vibration activity, called coincidence detection, which can further improve the detection probability and reduce the false-alarm rate. Vibration detection performance can be optimized by adjusting $M_{p}$, $M_{g}$, M and K in the signal pre-processing procedure. After setting the parameters, the data matrix collected in real-time by the system injecting light pulses periodically into the optical fiber can generate the corresponding grayscale images through numerical mapping for later image pre-processing and target detection.

### 3.3. Median Filtering and Pseudo-Color Processing

As described in Section 3.2, one array of detection data was obtained from $M_{p}$ detection pulses, and the vibration spatio-temporal data matrix can be generated during continuous system detection. While there is still a certain probability of false alarm after effectively improving the detection probability, discrete noise will appear if the spatio-temporal image is generated directly. Before obtaining the spatio-temporal images, the grayscale images generated from the data matrix need to be pre-processed to reduce the impact of image noise on later multi-target detection, enabling the targets in the images to be better recognized, and the classification accuracy to be improved [28]. The system uses median filtering to smooth the image pixel values to achieve the effect of denoising [29]. The median filter takes the median value of adjacent pixels as the output of the current pixel to suppress high-frequency noise before image detection. Afterward, the data image is processed using the pseudo-color image fusion method with low computational cost to achieve image enhancement by assigning different colors to the gray levels in the image [30]. The fused image can enhance the performance of object segmentation, feature extraction, and target object detection [31]. The system generates in real-time the final spatio-temporal images to be detected after the process described in Section 3.2 and Section 3.3.

### 3.4. Vibration Data Collection

Fiber optic sensing technology overcomes the shortcomings of high-speed rail tracking circuits easily damaged by lightning. Not only can it obtain the running status of the train in real-time [32], but it also can play a well-assisted role in high-speed railway perimeter intrusion detection [33]. The abnormal vibration to be detected by the Φ-OTDR high-speed railway perimeter security warning system originates primarily from construction machinery, manual excavation, walking near fences, climbing fences, airflow induced by natural wind, or high-speed rail passing by. The first three types of early warning need to be monitored by buried optical fiber cable. The last two types are monitored by optical fiber cable hung on razor barbed wire, where fence climbing needs to be an alarm in real-time, and vibration caused by airflow does not require an alarm.

The fiber optic sensing device uses a narrow linewidth (~1.69 kHz) laser at 1550 nm with a typical output power of 13 dBm. Optical frequency shifts 200 MHz by controlling the acousto-optic modulator with 50 ns pulse width. Detailed parameters of the device signal processing are as follows: the optical pulse repetition frequency is 5 kHz, the sampling rate is 250 MS/s for the DAQ, $M_{p}$ as the number of samples in one single sweep is 1000, the noncoherent integration samples number M is 880, and the pulse canceller interval $M_{g}$ is 100. One pixel corresponds to a distance dimension of 0.4 m and a time dimension of 0.2 s in the generated spatio-temporal vibration image. Different threshold adjustment factors and K values were set at different monitoring areas of the security perimeter of the HSR station to reduce the effect of coherent noise.

The system collected vibration data images of different classes at four ground detection areas and multiple razor barbed wire detection areas in 30 consecutive days, and vibration data images of changes over the spatio-temporal domain were generated in real-time after signal pre-processing and image processing. The fence climbing and airflow activities were sensed by monitoring the optical fiber cable hung on razor barbed wire. Three kinds of activities nearby the fence, namely construction machinery, manual excavation, and walking, were sensed by monitoring the optical fiber cable laid about 20 cm underground and parallel to the fence at a distance of 0–1 km from the starting position of the fiber optic. The experimenter collected data images by walking within the warning area directly above the buried optical fiber cable nearby the fence, and also simulated construction machinery and manual excavation activities by compacting the ground using a pneumatic hammer and hitting the ground using a shovel at a distance of about 0.4 m perpendicular to the buried optical fiber cable laying direction, respectively. Vibration data for the above 3 types of activities were collected by the real activities of the experimenters. Razor barbed wire binding optical fiber cable was shaken with a wooden stick to simulate climbing over the fence. The experimenters used a larger force with a wooden stick in the actual data collection to simulate a real climbing event in order to try to achieve a more realistic simulation. Considering the real-time requirement of the alarm and the authenticity of vibration activities, the duration of a construction simulation activity is 3–6 s, the duration of a person walking simulation activity is 3–15 s, and the duration of a simulation activity of climbing over the fence is 5–18 s. Furthermore, the razor barbed wire vibration data caused by the airflow of the natural wind or HSR passing by was recorded continuously by the system for a variable duration.

**Figure 3:**
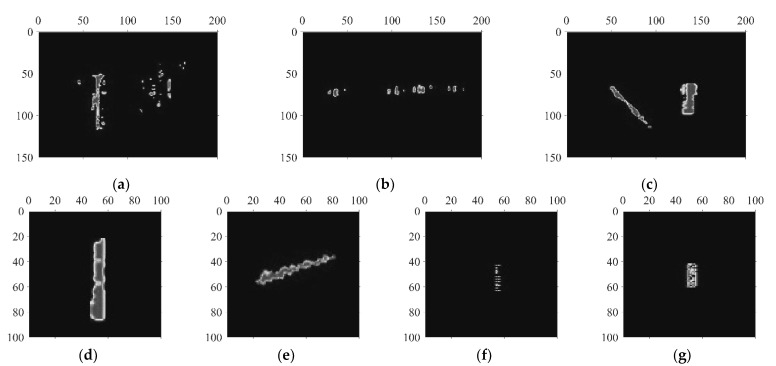
Grayscale images of different vibration events: (**a**) Natural wind; (**b**) HSR passing by; (**c**) Walking and climbing over fence simultaneously; (**d**) Climbing over fence; (**e**) Walking near fence; (**f**) Manual excavation; (**g**) Construction machinery.

**Figure 4:**
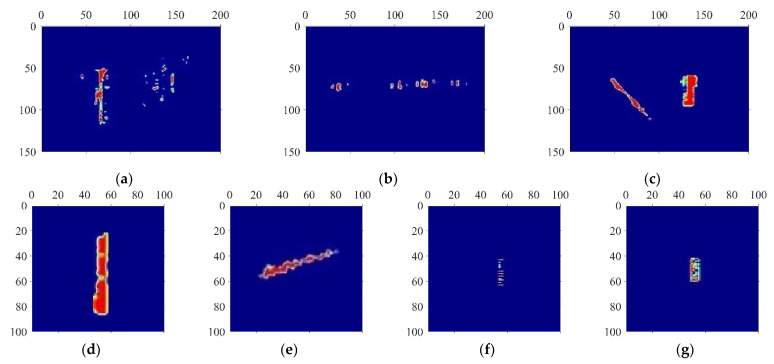
Data images of vibration events after pseudo-color processing: (**a**) Natural wind; (**b**) HSR passing by; (**c**) Walking and climbing over fence simultaneously; (**d**) Climbing over fence; (**e**) Walking near fence; (**f**) Manual excavation; (**g**) Construction machinery.

Figure 3 shows the grayscale image of the vibration signal generated by the signal matrix after median filtering. The seven grayscale images show the seven typical event manifestations of the five classes of vibration activities. It can be visualized that the spatio-temporal images can well illustrate the vibration events. The width and height pixel values of the images are marked sequentially, where width indicates the vibration distribution along the fiber distance dimension and height represents the vibration duration. Figure 4 shows the pseudo-color images of the grayscale photographs of the seven different vibrational events after image enhancement processing to visually highlight the effect of pseudo-color processing.

## 4. Experimental Image Dataset and Methodology

The data pre-processing procedure can reduce the false-alarm rate and detect the location and time of vibration occurrence. However, in the actual detection environment of HSR perimeter security, there will still be vibrations from the natural wind or from the HSR passing by, thus resulting in false alarms. Such vibrations not within the alarm classes require an improved YOLO target detection model training with the customized dataset to recognize different patterns and remove non-essential alarms.

### 4.1. Image Data Augmentation

Convolutional neural network target detection systems have superior performance compared to traditional computer vision algorithms. However, overfitting problems can lead to performance degradation, which can typically be avoided by regularization methods [34] or increasing the amount of data. Data augmentation methods can increase the number of additional samples in the dataset, thus improving the generalization ability of the system, and effectively enhancing its performance for small target detection [35], including rotation, horizontal flipping, resizing, hue, saturation alteration, etc.

### 4.2. Dataset Generation

Four thousand three hundred eighty-one vibration data of 5 classes at different times and locations were collected by the method in Section 3.4.

The image dataset size was increased using the image data augmentation techniques in Section 4.1 to improve the robustness and generalization ability of the system, and each spatio-temporal domain vibration image generated a corresponding dataset image of size 608 pixels × 608 pixels to finally build a dataset of HSR perimeter security fiber optic sensing vibration images and generate the corresponding YOLO format annotation files. A total of 8069 images were eventually used for the experiments, as shown in Table 1, and the dataset was randomly assigned into a training set, test set, and validation set in accordance with the ratio of 8:1:1. In addition, the five vibration activities are divided into three alarm event types according to the practical application. Vibrations caused by airflow are natural phenomena and occur frequently, and such events do not require an alarm. When there is human activity or mechanical activity in the monitoring area near the fence, the detection system needs to send an early warning message to alert security personnel to the vibration changes in the area. As a critical monitoring vibration activity, climbing over the fence activities need the real-time alarm to inform personnel to rush to the scene to deal with such violations.

### 4.3. Target Detection Meta-Architecture Analysis

The target detection module is an important and integral part of the entire system. Its primary accomplishments include detection of target number in spatio-temporal data images, recognition of vibration pattern, and annotation of the corresponding bounding box, i.e., the position of the target in the image. This subsection focuses on the training process of the YOLO target detection model and the factors affecting the recognition results to provide a basis for the improvement of the multi-target detection model.

The YOLO target detection meta-architecture training process can be divided into three parts:

1. Backbone and Neck are the most dominant feature extraction networks of the model, and these parts extract feature maps at different scales by linear and nonlinear operations;
2. The detection Head contains multiple scales to be detected, the number of which depends on the number of different scales of the feature map after the aforementioned downsampling feature extraction, and the feature map width and height on each scale depends on the number of times the original spatio-temporal image has been downsampled;
3. The loss function defined by the YOLO detector includes three types of loss values: target confidence loss, localization loss [36,37], and classification loss. The difference between system detection results and prior knowledge in target detection and localization can be judged by these three types of loss scores.

**Figure 5:**
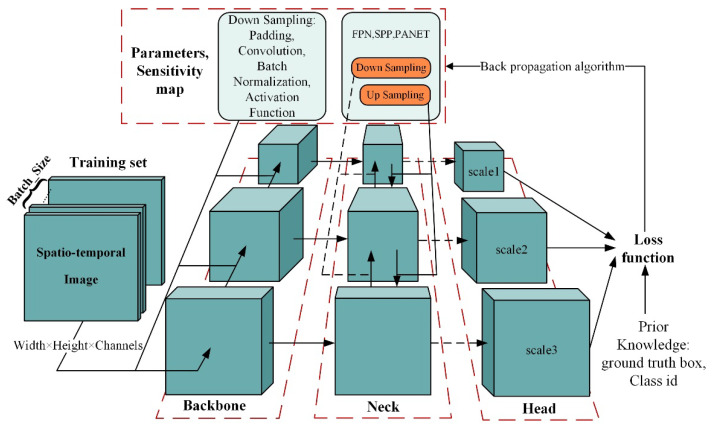
YOLO target detection meta-architecture training process.

As illustrated in Figure 5, the spatio-temporal vibration data images in the training set are fed into the Backbone. The feature maps at different scales are extracted from the Backbone and Neck sections by the convolutional unit to complete the downsampling. It is worth mentioning that the activation function in the convolutional unit allows the convolutional network to extract nonlinear features while avoiding the vanishing gradient problem. Currently, three activation functions are used in typical convolutional units frequently. Leaky-ReLU is simple and easy to implement with low computational cost. Unlike the Leaky-ReLU function, the non-monotonic and smooth nature of Swish [38], which is defined as $f\left(x\right)=x\cdot sigmoid\left(x\right)$, provides some robustness to different initialization and learning rates, and it is beneficial for model optimization and generalization ability, further improving the classification accuracy. Similar to Swish, Mish [39] is a smooth and non-monotonic activation function. Although Mish can improve the detection accuracy of neural networks, it increases the training time. Feature maps at different scales will enhance the discriminability and robustness of target features in the Neck section by expanding the receptive field. Furthermore, this part will accomplish feature propagation at different scales through a path aggregation network consisting of several bottom-up paths and several top-down paths to achieve feature enhancement [40,41]. In addition, CSPNet [42], Focus module, and Spatial Pyramid Pooling (SPP) network [43] are among the feature extraction network optional modules for feature enhancement.

The detection Head subsequent to the feature extraction network maps the depth of the feature map at different scales to $\left(classes+5\right)\times 3$, where classes indicates the number of types to be detected, 5 refers to an object confidence value and four values represent the location of the bounding box, and 3 denotes the number of prior anchor boxes at each scale. In this paper, the depth of the feature map is 30 for each scale.

The target confidence loss, localization loss, and classification loss are obtained by calculating the difference between the results of the detection system and the prior knowledge, updating the sensitivity map and the parameters of each convolutional layer of the detector using the sum of these three types of losses and the backpropagation algorithm, iterating the above steps until convergence, and until the detector can meet the detection requirements on the test set, thus completing the entire training process.

It should be remarked that the localization loss can be chosen from different Intersection over Unions (IoUs): Generalized IoU (GIoU) [36], Distance-IoU, and Complete IoU (CIoU) loss [37]. Among them, the CIoU loss considers the three geometric factors of overlap area, central point distance, and aspect ratio, thereby leading to faster convergence and better performance.

Based on the above analysis, the application of different feature extractors is based mainly on the complexity of the detection problem itself. The feature extraction network structure affects the final detection performance during the training process. The long-distance fiber optic vibration monitoring system needs to monitor the abnormal vibration at each region in real-time and reduce the false-alarm rate of the system at the same time. Therefore, it is required to consider both the real-time ability of system inference and the requirement of multi-target detection accuracy in designing the feature extraction network of the system.

### 4.4. Network Construction

In order to achieve high-precision detection of vibration activity in real-time using Φ-OTDR, four different types of feature extraction networks designed for fiber optic vibration images were implemented based on the analysis of the feature extraction network in Section 4.3, as presented in Table 2. The improved models were trained and tested in experiments using the image dataset in Section 4.2.

The design of the feature extraction network depends mainly on the complexity of the detection problem itself, and the main factors to be considered in the design include the type and number of feature extraction layers. Generally, the deeper the network is, the easier it is to obtain more abstract features. Yet, at the same time, it will increase model complexity and the number of parameters, which will affect the computational speed and real-time recognition ability of the system. The relationship between detection accuracy and model complexity can be analyzed by choosing different feature extraction components and different combinations of approaches in the feature extraction networks described above, and a suitable improved model can be selected using the evaluation metrics specified in Section 4.5. The feature extraction network consists of a combination of different feature extraction modules in the Backbone and Neck, where the CBA block contains a convolutional layer, a batch normalization layer, and an activation function, and the CSPNet block consists of two paths that are connected together by a concat layer to form a feature map after the features are extracted by the CBA block and several ResBlocks.

**Figure 6:**
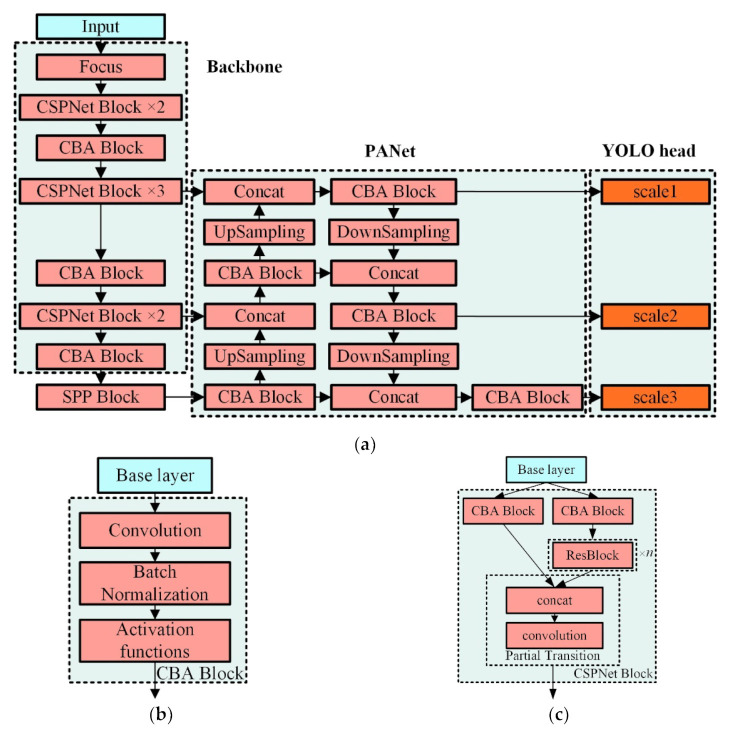
Feature extraction network and main components: (**a**) Improved YOLO-A30 network architecture; (**b**) CBA block component; (**c**) CSPNet block component.

The improved YOLO-A30 network architecture is shown in Figure 6. YOLOv3-tiny and YOLOv3 [18] were trained as control groups to compare the prediction performance of different feature extraction networks using the YOLO meta-architecture.

### 4.5. Evaluation Metrics

Precision, recall, F1 score, mean average precision (mAP), inference time, and parameters (M) were used as the evaluation metrics to evaluate the detection effectiveness of different networks in a comprehensive manner.

The definitions of precision, recall, F1 score, average precision (AP), and mean average precision (mAP) are expressed by Equations (8)–(12). True positive (TP) indicates the amount of target data where the positive class of the outcome of the model detection is consistent with the actual class in the dataset. False positive (FP) represents the amount of data where the positive class of the outcome of the model detection is inconsistent with the actual class in the dataset, i.e., the number of false detections. False negative (FN) then denotes the number of target data in the dataset with actual classes but detected by the model as other classes, i.e., the number of missed detections. Precision and recall indicate the confidence of the model in target detection and the ability of the model to detect all targets, respectively. The precision and recall metrics of the model can be comprehensively evaluated using F1. Under a fixed IoU, the given target will get different precision and recall values according to different confidence, and the continuous curve generated by interpolating the precision and recall values is the precision-recall (PR) curve. Average precision (AP) shows the comprehensive performance of the model at different confidences by the area under the curve (AUC) on the PR curve for a given target class, and a higher AP value represents better model detection performance. Each IoU corresponds to a different AP, and the AP@.5 corresponds to the AP when IoU takes 0.5, AP@[.5:.95] corresponds to the average AP for IoU from 0.5 to 0.95 with a step size of 0.05. The mean average precision (mAP) evaluates the mean of the average precision of the model on all detection classes. Eventually, the inference time describes the time required by the model for single image detection. Usually, the fewer model parameters, the smaller the GPU memory consumption, the shorter the inference time, and the more it can be implemented on embedded devices.

(8) $$\mathrm{precision}\;=\frac{\mathrm{TP}}{\mathrm{TP}+\mathrm{FP}}$$

(9) $$\mathrm{Recall}=\frac{\mathrm{TP}}{\mathrm{TP}+\mathrm{FN}}$$

(10) $$F_{1}=2\times \frac{\;\mathrm{Precision}\;\times \;\mathrm{Recall}\;}{\;\mathrm{Precision}\;+\;\mathrm{Recall}\;}$$

(11) $$\mathrm{AP}\left[\mathrm{class}\right]=\sum_{i\;\in \;\mathrm{confidence}\;}{\mathrm{precision}}_{i}\left[\mathrm{recall},\mathrm{class},\mathrm{iou}\right]$$

(12) $$\mathrm{mAP}=\frac{1}{N}\sum {\mathrm{AP}}_{i}$$

## 5. Experimental Procedure

### 5.1. Experimental Setup

The phase-sensitive optical time-domain reflectometer is used as a vibration signal collector in the monitoring area, as described in Section 2.1. The pulsed light is amplified by EDFA and injected into the fiber through an isolator and circulator. The backscattered light is detected by a photodetector, and the collected raw data is formed into spatio-temporal images after signal pre-processing, median filtering, and pseudo-color technique. The data images of different vibration events are collected, and the ultimate dataset is formed through data augmentation. The parameters for equipment data acquisition and pre-processing, the method of single-mode fiber deployment, and the method of data acquisition are specified in Section 3.4.

**Figure 7:**
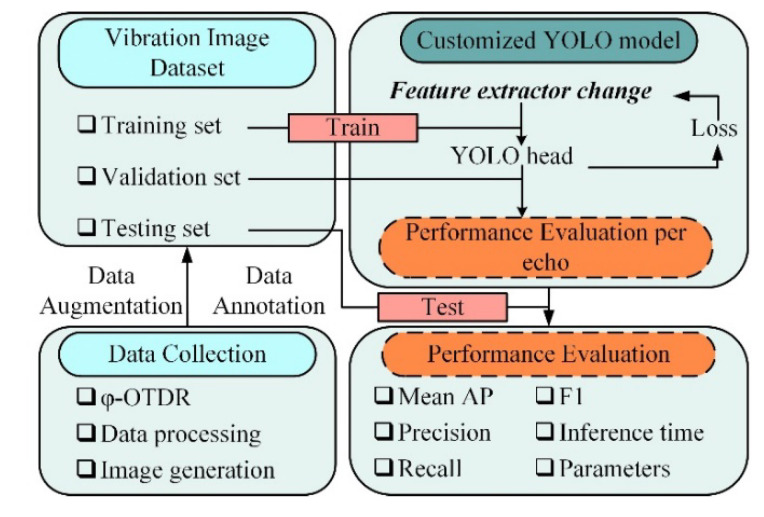
Experimental procedure of the proposed optical fiber sensing vibration identification method based on deep learning.

This study combines different network modules based on the YOLO algorithm framework to implement four feature extraction networks, using the customized dataset to train the models. The specifics of the feature extraction networks are elaborated in Section 4.4. The prediction performance of the improved models is compared comprehensively using the previously described evaluation metrics. The experimental procedure is shown in Figure 7. Considering the practicality and computational complexity, the feature extraction networks in this paper use the Swish activation function, CIoU localization loss and YOLOv3 detection Head, and combine different feature extraction components to achieve YOLO model improvement. The experimental contents of the fiber optic sensing vibration recognition system contain the following:

1. The YOLO target detection models composed of different feature extraction networks are trained using the training set, the training effect is tested using the validation set, and the optimal model in each type is selected; the performance metrics of the selected models in each type as described in Section 4.5 are measured using the test set, and the one with a better balance of various metrics is chosen as the final improved model;
2. Various combinations of vibration type data in the test set are selected to simulate different mixed vibration activities to test and discuss the qualitative detection results of the final improved model;
3. The metrics of the final improved model and other SOTA models arere counted using the test set and comparisons are made.

### 5.2. Network Training

Using YOLOv3-tiny and YOLOv3 as control groups, the corresponding performance metrics are obtained by varying the feature extraction network of the YOLO meta-architecture. The training set is used for model parameter updates, the sum of the three types of losses described in Section 4.3 is calculated for each iteration of the optimization process during the experimental training, which in turn updates the model parameters by the back-propagation algorithm, and the model performance metrics are counted using the validation set at the end of each epoch during the training process, while the system hyperparameters are kept consistent. Considering the model generalization ability and overfitting problem, each feature extraction network is trained at least three times and 100 epochs are run each time, from which the model with the best-combined performance of AP@.50 and AP@[.5:.95] in the validation set is selected, and the model performance is tested by the test set. The model training process was run on an Ubuntu 18.04 operating system with an NVIDIA GTX 3090 GPU with 24-GB onboard memory, accelerated by CUDA (version 11.1) and cuDNN (version 8.0). YOLO meta-architecture is based on Python (version 3.8) and the PyTorch library (version 1.8.0) released by Facebook AI.

**Figure 8:**
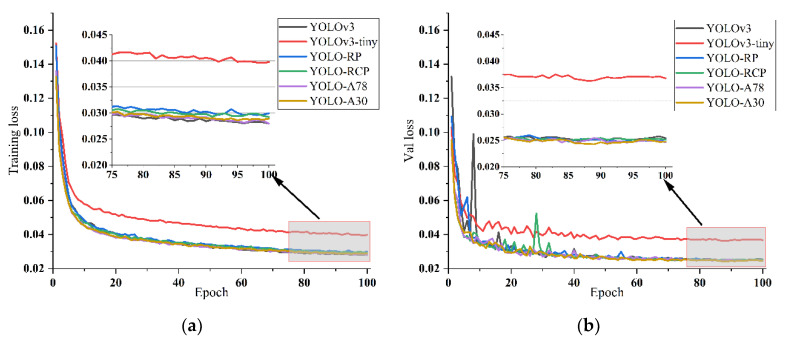
Training loss and validation loss of different models: (**a**) Training loss of six experimental models; (**b**) Validation loss of six experimental models.

Figure 8 shows the training loss and validation loss of different feature extraction networks after 100 epochs of training. The training losses decrease gradually during the training process, which indicates that the networks learn model parameters from the training set effectively without overfitting. YOLOv3 and the four types of improved models each have significantly lower losses than YOLOv3-tiny, with the training loss ranging around 0.03 and the validation loss ranging around 0.025. It is observed that although the difference in training loss between YOLO-A30, YOLO-A78, and YOLOv3 is slight, YOLO-A30 validation loss is much lower than the other models, which reveals that YOLO-A30 performs better than the other models in the validation set.

**Figure 9:**
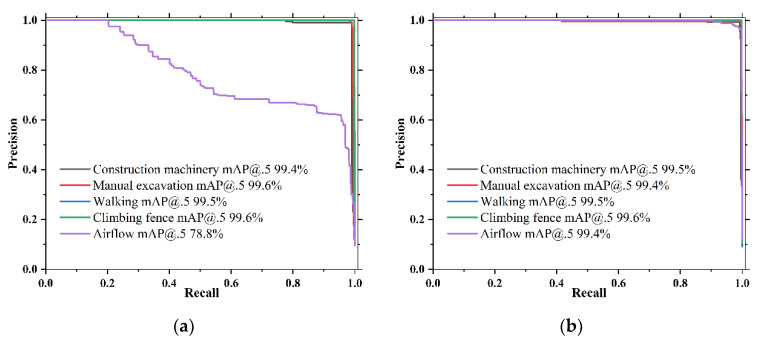
PR curve with IoU threshold of 0.5: (**a**) YOLOv3-tiny; (**b**) YOLO-A30.

Figure 9 shows the PR curves for YOLOv3-tiny and YOLO-A30 with an IoU threshold of 0.5. The AP@.5 values for the five detection events are represented by the area under the corresponding curves. It can be found from the PR curves that the detection performance gap between the improved YOLO-A30 and the control YOLOv3-tiny on airflow events is significantly larger than for the other activities. Other performance metrics of the models can be compared in Table 3.

Table 3 evaluates the prediction performance, the number of parameters, and the inference time of each model from seven metrics. For HSR perimeter abnormal vibration monitoring, fence climbing is the primary monitoring activity, which requires no missing alarms and high confidence of alarm. Vibration caused by airflow does not need to be alarmed, whereas due to the frequent commuting of high-speed rail and the windy field environment in which the high-speed rail line is located, the false-alarm rate of the system will be too high if the airflow cannot be identified precisely. However, the low recall of airflow can instead be considered automatic filtering of such events. Accordingly, while evaluating the overall performance, the F1 score of the fence climbing activity and the precision of the airflow event should be highlighted.

In the precision column, YOLO-A30 has the highest precision of 99.2%, which is 0.4% and 0.6% higher than YOLO-A78 and YOLOv3, which have more parameters, respectively. Its precision is the highest in the events of construction machinery, walking, and especially airflow, which is 0.5% and 36.7% higher than YOLOv3 and YOLOv3-tiny, respectively. Although YOLO-RP achieves 100.0% and 99.9% precision for manual excavation and fence climbing, its precision for airflow is only 86.4%.

The recall represents the probability that the model does not have missed detection, and all models achieve 100.0% in walking and fence climbing; for the crucial monitoring of fence climbing, especially, it is essential to achieve low missed-detection rate for the practical application of the model. For airflow events that do not require an alarm, the recall rate does not affect the system performance, so the effect of airflow recall should be removed when discussing the recall metrics. After removing the effect of airflow, the average recall of YOLO-A30 is 99.8%, reaching the highest value along with YOLO-RCP, which is higher than YOLOv3-tiny and YOLOv3 by 0.2% and 0.1%, respectively.

The F1 score can comprehensively evaluate the precision and recall of the model for detection activities, and the F1 score of YOLO-A30 is the highest at 99.2%. The F1 scores for construction machinery and walking activities are 99.1% and 99.8%, respectively. The F1 score for fence climbing is obtained in YOLO-RP with a maximum of 99.9%, which is 0.1% higher than that of YOLO-A30.

Focusing on the columns mAP@0.5 and AP@[.5:.95] with reference to YOLO-RP, YOLO-RCP, and YOLO-A30, although the number of parameters decreases in sequence, the mAP can be significantly improved with the addition of different components of the feature extraction network. The mAP@0.5 of YOLO-A30 is 0.1% and 4.1% higher than that of YOLOv3 and YOLOv3-tiny, and the AP@[.5:.95] is 1% and 9.4% higher, respectively, while the number of YOLOv3 parameters is 8.58 and 7.10 times greater than that of YOLO-A30 and YOLOv3-tiny. It can be noticed that using the same network components, increasing the number of parameters can improve the mAP value of the model by comparing YOLOV3 and YOLOV3-tiny performance metrics. However, there is a limit to the mAP gain from the increase in the number of parameters. YOLO-A30 and YOLO-A78 use the same network components, but with the increase in the number of parameters, mAP@.5 stays the same, and AP@[.5:.95] only increases by 0.1%. On balance, the YOLO-A30 with the fewest number of parameters performs better on the mAP metric.

When the system monitors abnormal vibration, the image target detection needs to achieve long-distance detection range and high timeliness, which is strongly related to the size of the spatio-temporal image to be detected and the inference speed. In this experiment, the length of the single image to be detected is 608 pixels, corresponding to the actual fiber distance of 243.2 m. Taking the YOLO-A30 with an inference speed of 1.8 ms as an example, the maximum multi-vibration events within 135.1 km can be detected in 1 s.

Comparing the inference speed of each model, it can be realized that the types of network components and the number of parameters used in the feature extraction network have an impact on the inference speed of the network. When designing a feature extraction network, one cannot just increase the number of network modules and parameters to improve detection performance while neglecting its impact on detection speed.

### 5.3. Qualitative Results

**Figure 10:**
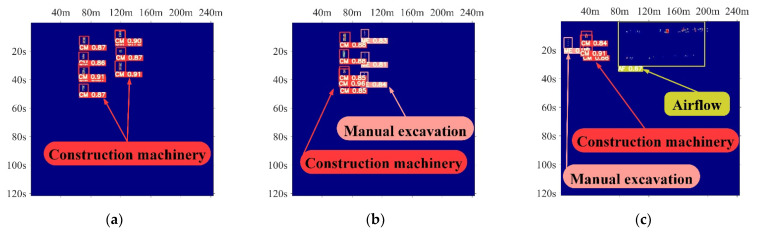

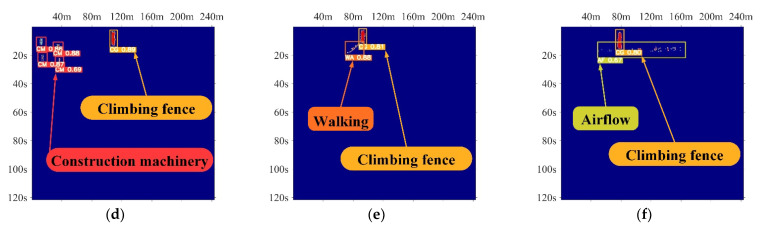
Effectiveness of six different mixed vibration events identification: (**a**) Multiple construction machines; (**b**) Multiple construction machines and manual excavation; (**c**) Airflow interference exists nearby the buried fiber optic site where the early warning activities occurred; (**d**) Climbing over the fence behavior and construction machinery activities occur in the same monitoring area; (**e**) People walking in the buried fiber optic area and climbing over the fence; (**f**) Person climbing over the fence during airflow interference.

The above metrics illustrate the performance of YOLO-A30 on the test set. Benefiting from the distributed nature of fiber optic sensing, the vibration signals generated by different activities occurring simultaneously at different locations of the fiber do not affect each other. The combinations for different vibration classes in the test set are picked to simulate six different kinds of mixed vibration events to visualize the results of multi-target detection of YOLO-A30 in specific scenarios. The test parameters are IoU > 0.5, Confidence > 0.4, as depicted in Figure 10. The image width represents the range of influence in the fiber distance dimension corresponding to the multi-vibration event in meters. The image height indicates the duration of the multi-vibration event in seconds. Although repeated detection occurs when the vibration signal is too dense in the buried fiber state, the system can effectively distinguish multiple vibration classes in the same detection range.

Figure 10a,b separately depict the two construction early warning activities detected by the system in the buried fiber state and their respective detection confidence. When multiple mechanical construction events occur in the monitoring area, YOLO-A30 can effectively identify the vibration activity class of the event and divide the multiple vibration activities. When two classes of construction activities occur simultaneously at different locations, the system will automatically distinguish the different classes of activities and label them by the bounding box.

Figure 10c–e display three scenarios where vibration is detected simultaneously in the two cases of fiber buried underground and hung on razor barbed wire. Due to the distributed nature of the fiber optic sensor, the system can still accurately detect construction events in the buried fiber state in the presence of airflow interference. Whether there is a continuous behavior such as people walking and climbing over the fence or construction vibration disturbances, the model can identify such a crucial monitoring activity as climbing over the fence.

Figure 10f illustrates the extreme case when airflow interference and fence climbing occur under the fiber optic hanging condition in the same area. The system can eliminate the interference and identify fence climbing activity to improve the overall detection performance.

### 5.4. Comparison with Other State-of-the-Art Models

**Figure 11:**
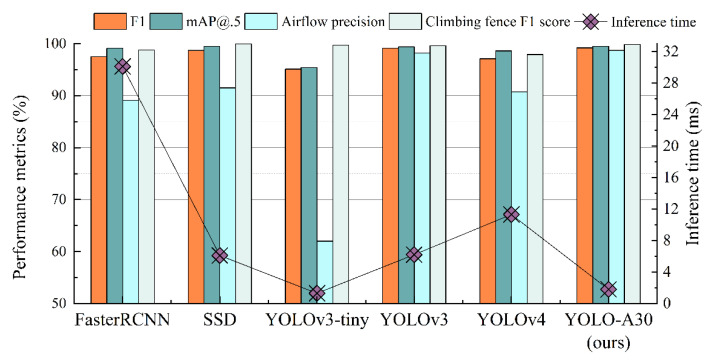
Performance metrics of YOLO-A30 and other SOTA models on focused activities.

The performance of YOLO-A30 is compared with other SOTA models on the dataset described in Section 4.2. The mAP@.5, F1 score, airflow precision, climbing fence F1 score, and inference time of 6 models, Faster R-CNN [14], SSD [17], YOLOv3-tiny, YOLOv3 [18], YOLOv4 [19], and the improved YOLO-A30, are shown in Figure 11, considering the high-speed rail application environment.

YOLO-A30 has the highest F1 score of 99.2%, indicating that it can detect the five classes of abnormal vibration activities in the dataset relatively accurately with a low missed-detection rate. F1 scores of YOLOv3 and SSD are only lower than YOLO-A30, each reaching 99.1% and 98.7%, both with excellent performance, but both are more than three times slower than YOLO-A30 in terms of detection speed, which means the longest distance these two models can monitor is much shorter than the monitoring distance of YOLO-A30. Over 95% mAP@0.5 are achieved for all models, with YOLO-A30 and SSD both reaching a maximum of 99.5%, 4.1% higher than the lowest, YOLOv3-tiny. The precision of airflow detection, which is the most frequent vibration interference event in the actual application environment for high-speed rail, is also noticed separately in the figure, and the precision of this event monitoring will directly affect the final false-alarm rate of the system. One explanation for the considerable variation in airflow precision across models is that airflow events do not have a fixed shape and aspect ratio in the spatio-temporal data image, which leads to particular difficulty in detection and requires a high feature extraction capability for the model network, which makes this metric a critical factor in differentiating model performance. The airflow precision of YOLO-A30 is 98.7%, which is 0.5% higher than YOLOv3 and significantly higher than other target detection models. The SSD reaches 100% in the fence climbing F1 score, which is 0.2% higher than the YOLO-A30, but it lags behind the YOLO-A30 in all other metrics; in particular, the differences in airflow precision and inference time are 7.2% and 4.3 ms, respectively. The inference time of Faster R-CNN [14] as a two-stage detection is 30.1 ms, which is much longer than other single-stage detection models, which also shows from one side that the single-stage detection model is more appropriate for long-distance monitoring requirements.

Overall, YOLO-A30 performs excellently in the main metrics compared to other SOTA models. It can avoid false alarms of fiber optic sensors due to airflow to a certain extent and detect five key monitoring events simultaneously, providing a possible solution for high-speed rail perimeter security automation.

## 6. Conclusions

In this paper, a real-time multi-vibration events detection method based on an improved YOLO model for Φ-OTDR optical fiber buried underground and hung on razor barbed wire dual detection environments is proposed for high-speed railway perimeter abnormal vibration detection, aiming to realize long-distance perimeter security automation and reduce system false-alarm rate and security cost. Based on the statistical characteristics of the acquired raw signals and the signal detection theory, it is shown how pulse accumulation, pulse cancellers, and coincidence detection can improve the detection performance of the system. Following this, the signal pre-processed data matrix is median filtered and pseudo-color processed to achieve feature enhancement and generate a spatio-temporal vibration image dataset with 8096 images of five vibration activities for training, validation, and testing. Improving the feature extraction network of the YOLO target detection model, specifically using different network components and network structures, aims to discover a deep learning architecture more appropriate for fiber optic vibration monitoring tasks in the application environment of the high-speed railway. The experimental results demonstrate that the improved YOLO-A30 model achieves a satisfactory balance in precision corresponding to the false-alarm rate of the system, recall related to the missed-detection rate of the system, and inference speed, which exhibits a better detection performance than other experimental models. This study illustrates that the method can automatically perform Φ-OTDR acquisition signal pre-processing and effectively extract vibration event features to identify abnormal vibration, which reduces the false-alarm rate in the practical application of distributed fiber optic sensing system and makes it possible to apply this method in high-speed railway perimeter security.

Future work will focus on increasing the detection distance of a single device through multi-channel or other Φ-OTDR techniques and optimizing the existing results, including validating model accuracy and performance in the actual scene, evaluating the feasibility, mining for more possible vibration events, and verifying the adaptation of the model to recognize similar events to improve the robustness and adaptability of the model, as well as developing embedded devices for practical applications.

## Figures and Tables

**Table 1:** Distribution of each vibration pattern in the described dataset.

Fiber Optic Laying Method	Activity	Activity Simulation	Amount of Collected Data	Collection Location	Duration of a Collection	Total Data Amount with Data Augmentation	Alarm Type
Optical fiber cable buried underground	Construction machinery	Pneumatic hammer	1357	0–1 km	3–6 s	2714	Early Warning
	Manual excavation	Shovel type construction	1051	0–1 km	3–6 s	2102	Early Warning
	Walking	People walking within the warning area nearby the fence	137	0–1 km	3–15 s	819	Early Warning
Optical fiber cable hung on razor barbed wire	Climbing over fence	Shaking Razor Barbed Wire	200	1–5 km	5–18 s	798	Real-time alarm
	Airflow	Natural wind or high-speed rail passing by	1636	1–5 km	Variable	1636	False alarm

**Table 2:** Six types of feature extraction networks composed of different network components.

Model		Backbone			Neck		Head	Number of Convolutional Layers
	Focus	ResBlock	CSPNet	SPP block	FPN	PANet	YOLO	
YOLOV3-tiny					√		√	13
YOLOV3		√			√		√	52
YOLO-RP		√				√	√	30
YOLO-RCP		√	√			√	√	30
YOLO-A30	√	√	√	√		√	√	30
YOLO-A78	√	√	√	√		√	√	78

**Table 3:** Models performance metrics statistics.

Model	Class	Precision (%)	Recall without Airflow (%)	F1 (%)	mAP@.5 (%)	mAP@[.5:.95] (%)	Parameters (MB)	Inference (ms)
YOLOV3-tiny	All	91.6	99.6	95.1	95.4	59.9	17.4	**1.3**
	Construction machinery	98.2	99.0	98.6	99.4	58.1		
	Manual excavation	99.0	99.2	99.1	99.6	58.1		
	Walking	99.4	100.0	99.7	99.5	75.2		
	Climbing fence	99.5	100.0	99.7	99.6	69.4		
	Airflow	62.0	95.7	75.2	78.8	38.9		
YOLOV3	All	98.6	99.7	99.1	99.4	68.3	123.5	6.2
	Construction machinery	97.2	99.0	98.1	99.3	62.8		
	Manual excavation	98.9	99.6	99.2	99.6	58.5		
	Walking	99.4	100.0	99.7	99.5	81.4		
	Climbing fence	99.3	100.0	99.6	99.5	71.1		
	Airflow	98.2	100.0	99.1	99.2	67.4		
YOLO-RP	All	96.7	99.7	98.0	98.8	68.6	20	1.8
	Construction machinery	97.8	99.0	98.4	99.5	64.0		
	Manual excavation	100.0	99.8	**99.9**	99.6	59.8		
	Walking	99.4	100.0	99.7	99.5	82.3		
	Climbing fence	99.9	100.0	**99.9**	99.6	73.2		
	Airflow	86.4	98.1	91.9	95.8	63.5		
YOLO-RCP	All	97.0	**99.8**	98.1	98.9	68.2	17.8	1.7
	Construction machinery	97.6	99.4	98.5	99.5	63.5		
	Manual excavation	99.6	99.8	99.7	99.5	60.5		
	Walking	99.4	100.0	99.7	99.5	82.2		
	Climbing fence	98.8	100.0	99.4	99.5	71.7		
	Airflow	89.7	96.9	93.2	96.1	63.3		
YOLO-A30	All	**99.2**	**99.8**	**99.2**	**99.5**	69.3	**14.4**	1.8
	Construction machinery	98.8	99.4	**99.1**	99.5	62.5		
	Manual excavation	99.5	99.6	99.5	99.4	59.3		
	Walking	99.6	100.0	**99.8**	99.5	83.5		
	Climbing fence	99.6	100.0	99.8	99.6	71.7		
	Airflow	**98.7**	96.9	97.8	99.4	69.6		
YOLO-A78	All	98.8	99.7	99.1	**99.5**	**69.4**	16.4	2.3
	Construction machinery	98.6	99.7	**99.1**	99.6	61.6		
	Manual excavation	99.0	99.2	99.1	99.5	59.6		
	Walking	99.5	100.0	99.7	99.5	82.3		
	Climbing fence	99.5	100.0	99.7	99.5	73.4		
	Airflow	97.6	98.8	98.2	99.2	70.2		

The key performance metrics with the best results are highlighted.

## Data Availability

The data presented in this study are available on request from the corresponding author.
